# Distress intolerance amplifies the effect of momentary anxiety on momentary cigarette craving among females who smoke cigarettes

**DOI:** 10.1016/j.addbeh.2025.108421

**Published:** 2025-06-30

**Authors:** Brianna R. Altman, Jacqueline E. Smith-Caswell, Andrew H. Rogers, Angelo M. DiBello, Jordan A. Gette, Ana M. Abrantes, Teresa M. Leyro, Samantha G. Farris

**Affiliations:** a Department of Psychology, Rutgers, the State University of New Jersey, New Brunswick, NJ, USA; b Department of Medicine, Jacobs School of Medicine and Biomedical Sciences, University at Buffalo, the State University of New York, Buffalo, NY, USA; c Department of Applied Psychology, Graduate School of Applied and Professional Psychology, Rutgers, the State University of New Jersey, Piscataway, NJ, USA; d Department of Psychiatry and Behavioral Sciences, University of Kansas Medical Center, KS, USA; e Department of Psychiatry and Human Behavior, Alpert Medical School of Brown University, Providence, RI, USA; f Behavioral Medicine and Addictions Research Group, Butler Hospital, Providence, RI, USA

**Keywords:** Anxiety, Distress intolerance, Craving

## Abstract

**Introduction:**

Distress intolerance (DI) is an emotional vulnerability factor implicated in the link between anxiety and cigarette craving, which may be particularly important for characterizing persistent smoking in females. However, there is a dearth of prospective work examining how DI influences momentary aspects of anxiety and cigarette craving. This ecological momentary assessment study explored the main and interactive effects of momentary anxiety and DI on momentary cigarette craving.

**Methods:**

Females (N = 50) who reported daily combustible cigarette use completed a daily monitoring study that involved momentary assessments of anxiety and craving across one menstrual cycle. Trait DI was assessed at baseline. Multilevel modeling was used to examine the simultaneous between- and within-person effects of momentary anxiety and the moderating influence of DI on momentary craving. We predicted that between- and within-centered momentary anxiety and DI, as well as their interactions, would be positively associated with momentary craving.

**Results:**

Results indicated that higher momentary anxiety, centered between- and within-person, predicted higher momentary craving. Although no main effect of DI on momentary craving was observed, we found a conditional effect of DI on within-person momentary anxiety in the prediction of momentary craving. The positive effect of within-person increases in momentary anxiety on momentary craving was amplified for those with higher DI relative to lower.

**Conclusions:**

High DI appears to amplify cigarette craving in the context of higher-than-average momentary anxiety. Future studies leveraging momentary methodologies are needed to further elucidate associations between anxiety, DI, and smoking processes to better understand contextual influences on DI. Such data can inform ideographic, in-the-moment interventions.

Despite reductions in smoking prevalence in the United States over the last fifty years (Cornelius et al., 2023), smoking prevalence remains elevated among individuals with anxiety (Loretan et al., 2022; Smith et al., 2018). The co-occurrence of anxiety and cigarette use exacerbates the risk of associated smoking harms, including greater dependence and withdrawal (Piper et al., 2010), and greater difficulty quitting smoking (Lawrence et al., 2011). Negative reinforcement models implicate anxiety as a precursor to cigarette initiation and maintained use (Leventhal & Zvolensky, 2015; McCarthy et al., 2010), whereby individuals smoke to reduce withdrawal symptoms, including elevated anxiety and stress, which (negatively) reinforces smoking behavior. Further, a significant body of empirical work highlights the prospective and bidirectional links between anxiety and smoking outcomes (e.g. onset, status, dependence; Fluharty et al., 2016; Garey et al., 2020). Sex differences exist in anxiety and smoking outcomes. Anxiety pathology is more prevalent in females (Mclean et al., 2012) and females are more vulnerable to smoking lapse following cessation (Smith et al., 2016), an effect which may be explained by affective vulnerability and craving. For example, although males and females differentially respond to smoking cues, females evidence greater craving, stress, arousal and negative affect following negative mood induction (Saladin et al., 2012). The relation between negative affect and craving also appears stronger for females (Erblich et al., 2022). While this work did not directly examine anxiety and cigarette craving relations, investigating this link in females could increase insight into processes that contribute to smoking persistence in this group.

Although anxiety and smoking outcomes demonstrate temporal instability (i.e. fluctuations over time (Shiffman, 2009b; Walz et al., 2014), these variables are often treated as static. Most published studies employ cross-sectional or experimental designs with single, retrospective assessments of anxiety and smoking outcomes. Such designs can obscure potential within-person heterogeneity in experiences and preclude examination of dynamic, covariation between anxiety and smoking outcomes prospectively. Ecological momentary assessment (EMA) studies that capture real-time smoking-related experiences can overcome these limitations and support testing between- and within-person hypotheses simultaneously (Shiffman, 2009a). Moreover, although experienced dynamically (Chandra et al., 2011; Perkins et al., 2009) and contextually (Dunbar et al., 2010), many studies that examine cigarette craving only include a single assessment point. Thus, EMA study of dynamic covariation between affective variables, including anxiety, and craving is warranted in females, as is a more in-depth inquiry into mechanisms of this association.

Distress intolerance, (DI) refers to an individual’s perceived or behavioral inability to withstand distress, including physical, cognitive, and emotional states (Leyro et al., 2010; Zvolensky et al., 2010) and represents a stable, trait-level transdiagnostic characteristic implicated in smoking (Veilleux, 2019) and anxiety (Keough et al., 2010). For individuals high in DI, cigarette smoking can serve as a low-effort strategy to regulate stress and/or negative affective states, which reinforces cigarette use over time (Leventhal & Zvolensky, 2015). For example, higher DI when measured behaviorally (i.e. persistence on several distressing tasks) is associated with increased negative affect on quit day and early lapse, relative to lower DI (Abrantes et al., 2008). Given that heightened craving may precede cigarette use (Gass et al., 2014), granular inquiry into trait DI’s role in dynamic, momentary links between anxiety and craving is warranted; nevertheless, limited research on this topic exists. One prior EMA study demonstrated that trait DI was correlated with between-person, high arousal negative affect (i.e. a composite variable of momentary assessments of being anxious/-worried, afraid/scared, and angry/frustrated; Sandel-Fernandez et al., 2023). A second relevant fourteen-day EMA study found evidence that a behavioral index of trait DI (i.e. a mirror tracing task) moderated the link between daily hassles (i.e. experiences of daily irritants, including relationships, occupational stressors, and financial concerns) and cigarette craving following a smoking cessation attempt (Volz et al., 2014). However, this work did not consider individuals’ *emotional* reactions to these stressors. Momentary emotional experiences, like anxiety, can be viewed as essential context for activating the vulnerability of high trait DI on smoking outcomes.

The present study sought to examine the between- and within-person effects of momentary anxiety, and the moderating influence of trait DI, on momentary cigarette craving in females who smoke cigarettes daily. We leveraged EMA to examine the nuanced and dynamic associations among these variables. Our hypotheses were as follows: first, we expected that between- and within-person deviations in anxiety would positively predict cigarette craving. Specifically, we expected that overall elevated anxiety (i.e. between-person effect) and momentary elevated anxiety (i.e. within-person effect) would predict greater cigarette craving. We also anticipated that those with higher trait DI would endorse higher momentary cigarette craving. Lastly, we hypothesized that trait DI would interact with both between-person and within-person deviations in momentary anxiety to predict craving, such that the associations between momentary anxiety indexed between people (i.e. between-person) and deviations in momentary anxiety (i.e. within-person) and craving would be strongest for those higher in DI.

## 1. Method

### 1.1. Participants

Participants (N = 50) were non-treatment seeking females who endorsed daily cigarette use and were recruited for an NIH-funded parent study, Project SHE, a prospective, daily monitoring study examining links between ovarian hormones, anxiety and naturalistic smoking behavior (Farris et al., 2023) . Although the primary parent study was designed to focus on the female menstrual cycle (Farris et al., 2025), that was not considered in this secondary data analysis study. Inclusion criteria included: (1) age between 18–40 years; (2) daily use of ≥ 5 cigarettes per day; (3) daily smoking for at least one year; (4) verification of smoking status via ≥ 10 ppm expired carbon monoxide (CO); (5) normal menstrual cycle (i.e., length of 25–35 days) that did not regularly vary in length month-to-month by ≥ 7 days. Exclusion criteria included: (1) current use of smoking cessation treatments; (2) use of hormonal birth control; (3) positive urine pregnancy test; (4) within 3 months postpartum; (5) breast feeding within the last three months; (6) history of medical conditions or current use of medications that have a known effect on ovarian hormones/menstrual cycle; (7) DSM-5 alcohol or substance use disorder (severe); (8) DSM-5 current eating disorder or BMI < 18; (9) Evidence of psychiatric instability (i.e., psychotic symptoms, mania, serious suicidal or homicidal ideation); 10) social instability likely to hinder adherence to home-based study protocol; 11) non-English speaking; or 12) inability to provide written informed consent.

### 1.2. Procedures

The study was conducted at a large university in the northeastern United States and all procedures were approved by the university’s Institutional Review Board. Participants were recruited through flyers locally and through an online advertising agency to draw a broader sample. Participants were telephone-screened for eligibility and potentially eligible participants were scheduled for an in-person baseline assessment. Participants provided written informed consent during the baseline assessment. Eligible participants were invited to participate in an at-home daily monitoring protocol that took place over the course of their menstrual cycle. They completed an orientation visit that occurred either on the same-day or day-following the baseline assessment. During the orientation visit, research staff reviewed the daily EMA protocol and allowed for practice.

The EMA protocol was programmed and administered through MetricWire, a web-based platform with an associated mobile app downloaded onto participants’ smartphone devices. MetricWire is a secure, private and HIPAA compliant platform. The daily EMA protocol included two time-based reports (morning, bedtime) and three signal-contingent (random) reports throughout waking hours, all lasting between three to five minutes each. Research staff monitored compliance daily and proactively worked with participants to ensure adequate compliance.

Participants were compensated $30.00 for completion of their baseline assessment. Compensation for the EMA protocol was based on compliance with participants earning $1.00/day for completing both morning and bedtime reports as well as $2.00 a day for completing at least two out of three random reports. Possible compensation for the aspects of the protocol in this study was $21 per week based upon compliance. Total possible compensation for all aspects of the parent study are detailed elsewhere (see Farris et al., 2023).

### 1.3. Measures

#### 1.3.1. Baseline assessment

Participants provided demographic information and completed a structured clinical assessment of psychiatric conditions and an initial Medical History Form to assess fit for the study. Participants completed a Carbon Monoxide (CO) Analysis, using the Vitalograph Breath CO carbon monoxide monitor, to confirm smoking status and a pregnancy test to confirm non-pregnant status.

The *Smoking History Questionnaire* (SHQ; (Brown et al., 2002) was administered to assess smoking history and patterns of self-reported use. The SHQ is a 30-item measure including items assessing for age of smoking initiation, smoking frequency, and years of daily smoking.

The *Fagerstrom Test for Cigarette Dependence* (FTCD; Fagerström & Fagerström, 2011) is a 6-item measure with high test–retest reliability used to assess cigarette dependence (Pomerleau et al., 1994). Item scores are summed to give a total score from 0 to 10 with higher scores indicative of greater dependence. Internal consistency of the FTCD items was a = 0.712.

*The Depression Anxiety Stress Scales-21* (DASS-21; Antony et al., 1998) is a 21-item self-report assessment comprised of three subscales designed to differentiate features of depression, anxiety, and stress. Items are answered from 0 (“Did not apply to me at all”) to 3 (“Applied to me very much or most of the time”) in regard to the past week. The anxiety subscale was used as a model covariate to evaluate the effects of DI above and beyond trait anxiety. Internal consistency of subscale items was a = 0.723.

The *Distress Tolerance Scale* (DTS; Simons & Gaher, 2005) is a 14-item, self-report assessment of perceived ability to tolerate distress generally. Example item statements include: “Feeling distressed or upset is unbearable to me,” and “I am ashamed of myself when I feel distressed or upset.” Items are answered on 5-point Likert-type scales ranging from 1 (*strongly agree*) to 5 (*strongly disagree*). Items are summed and a mean score is computed with higher scores reflecting lower distress intolerance and lower scores reflecting greater distress intolerance. Internal consistency of the DTS items was a = 0.917.

#### 1.3.2. EMA assessment

##### Anxiety.

Momentary anxiety was measured at all random reports throughout the day. Momentary anxiety was assessed with a single-item (“How anxious do you feel right now?”) ranging from 0 to 10 with higher scores reflecting greater momentary anxiety.

##### Cigarette Craving.

Momentary cigarette craving was measured with a single-item assessment (“How strong is your urge to smoke?”) ranging from 0 to 10 with higher scores reflecting greater momentary craving. Craving was assessed during all random reports.

##### Cigarettes Per Day.

Each morning, participants reported their previous day cigarette consumption. Cigarettes per day were then lagged to align with intended day and used to control for the effect of cigarette consumption in all models.

### 1.4. Data analytic plan

Analyses were conducted with SAS version 9.4. Examination of missing data revealed no systematic patterns of missing data, in line with a Missing at Random assumption, consistent with a multi-level modeling (MLM) approach. Prior to analyses, descriptive statistics and bi-variate correlations among variables were examined. Data were modeled using MLM to account for the nested data structure: assessments within days (level 1), days within person (level 2), persons (level 3). MLMs with random intercepts were fitted using SAS PROC MIXED as these models are recommended for computing unbiased estimates (Gabrio et al., 2022). Our primary aim was to examine the effects of momentary anxiety, centered within-person (i.e. within-person deviations in momentary anxiety assessments; level-1) and between-persons (i.e. comparisons between participant’s average momentary anxiety levels; level-3) on concurrent (same moment) cigarette craving, moderated by trait DI (level-3). Same day number of cigarettes (level-1), between-person baseline anxiety (level-3), and baseline nicotine dependence (level-3) were included in all models as covariates, given their associations with cigarette craving (Chandra et al., 2011; Donny et al., 2008; Heckman et al., 2013). This was done to isolate the effects of our independent variables (i.e. between- and within-person centered momentary anxiety, DI) on craving. We had no a priori hypotheses regarding the effect of within-day anxiety (level-2).

Data were modeled as follows: first, to determine the appropriateness of using MLM and determining the number of nested levels (using intraclass correlation (ICC)), an empty model (i.e., random intercepts for person and day only) was estimated for momentary cigarette craving. We determined a priori that ICCs of 0.05 or greater would indicate significant variance accounted for by a given level and thus should be retained. Predictors of interest (i.e. momentary anxiety, DI) were centered based on our aforementioned study hypotheses. Following determination of number of levels to retain, momentary cigarette craving was regressed onto momentary anxiety and DI variables and their interactions.

Significant interactions were probed using post-hoc simple slope analyses. Specifically, tests of simple slopes were used to evaluate the association between anxiety and craving at high and low levels of distress intolerance (− 1 *SD* and + 1 *SD* on the DTS, respectively) to determine which effects were significantly different than zero (Cohen et al., 2003).

## 2. Results

### 2.1. Descriptives and EMA compliance

Participants (N = 50) were 32.4 years old on average (SD = 5.3) and predominantly identified as white (70.0 %) and non-Hispanic (96.0 %). Females smoked cigarettes for an average of 14.9 ± 5.9 years, smoked an average of 12.2 ± 5.3 cigarettes per day and had moderate levels of cigarette dependence (Mean FTCD = 4.9 ± 2.2). Participants had a trait anxiety (DASS-anxiety) score of 4.2 ± 5.7 and a DTS score of 4.0 ± 0.9. There was no missing data for any model covariates (i.e. FTCD, DASS-anxiety, DTS scores).

Compliance with the EMA protocol was high. Participants completed at least one random report of momentary anxiety and craving on 92.5 % (n = 1288) and 92.3 % (n = 1286) of possible study days, respectively.

### 2.2. Preliminary analyses

The empty, intercepts only, model for momentary cigarette craving indicated an ICC of 0.08 at the day level, 0.43 at the between-person level, and 0.49 residual variance, suggesting that 8 % of the variance in momentary cigarette craving occurs between days, 43 % of the variance in momentary cigarette craving occurs between people, and 49 % of the variance in momentary cigarette craving varies at level 1, supporting the need to utilize a 3-level MLM (assessments, days, persons) for the substantive analyses. Thus, momentary anxiety was centered at the within-person deviations (level 1) and the between person level (level 3), and DI was grand mean centered and modeled at level 3.

#### 2.2.1. Main effects

We first examined a 3-level main effects model by regressing momentary cigarette craving on the following predictors: within-person momentary anxiety deviations at Level-1 and between-person momentary anxiety and DI at Level-3. The results from the main effects model reveal a significant effect of within-person momentary anxiety deviations at Level-1 (*b* = 0.346, β = 0.198, *se* = 0.026, *p* < 0.001) suggesting that higher levels of momentary anxiety than an individual’s own mean level are associated with greater momentary cigarette craving. Furthermore, there was also a significant effect for between- person momentary anxiety (*b* = 0.740, β = 0.383, *se* = 0.150, *p* < 0.001) suggesting that in aggregate, higher levels of momentary anxiety are associated with greater momentary cigarette craving. There was no significant effect for DI at Level-3 or any of the covariates (all *ps* > 0.083). Please see Table 1 for results of the full main effects model.

#### 2.2.2. Conditional effects

**Fig. 1.**
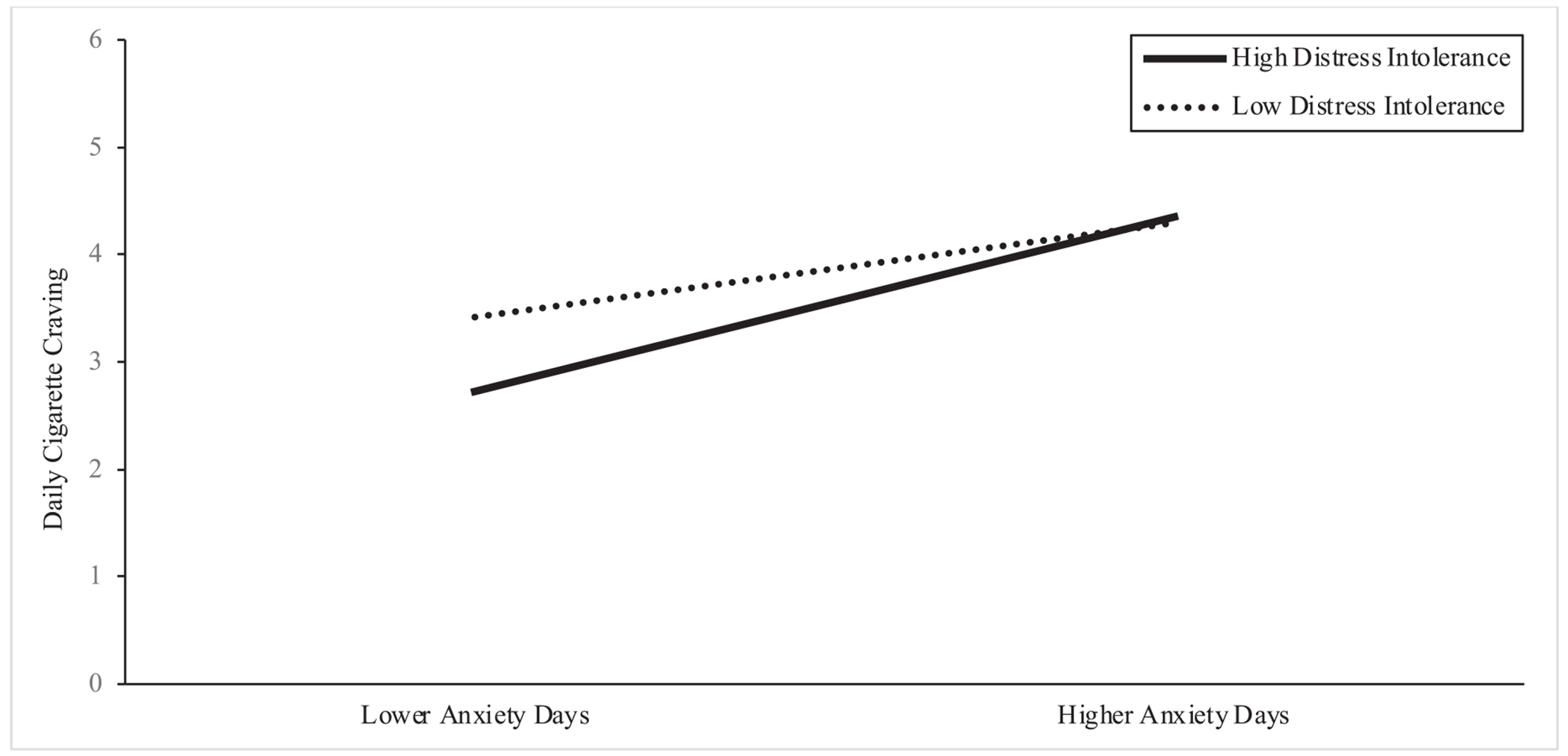
Simple slopes of the interaction between distress intolerance and anxiety on craving. Note: Lower Anxiety Days = Days in which participants’ anxiety is lower than their average momentary anxiety (i.e. centered within-persons); Higher Anxiety Days = Days in which participants’ anxiety is higher than their average momentary anxiety; High Distress Intolerance= Participants with lower scores on the DTS (i.e. 1 SD below the mean; low distress tolerance); Low Distress Intolerance= Participants with scores higher scores on the DTS (i.e. 1 SD above the mean; high distress tolerance). High distress intolerance appears to amplify the strength of the association between momentary anxiety and daily cigarette craving.

Second, we examined a conditional model which included the aforementioned main effects, covariates, and two two-way interactions: a cross-level interaction between within-person momentary anxiety deviations (level 1) and between-person DI (level 3), and an interaction between between-person momentary anxiety (level 3) and between-person DI (level 3) in order to test the moderating role of DI. The results from the interaction model reveal a significant cross level interaction between within-person momentary anxiety deviations at Level-1 and DI at Level-3 (*b* = −0.117, β = −0.056, *se* = 0.027, *p* < 0.001). Follow up tests of simple slopes reveal that the association between within-person momentary anxiety deviations and momentary cigarette craving is positive and stronger in absolute magnitude for those higher in DI (−1SD; *b* = 0.427, *se* = 0.032, *p* < 0.001) compared to those lower in DI (+1SD; *b* = 0.230, *se* = 0.037, *p* < 0.001). Stated differently, higher DI (compared to lower DI) amplified the momentary anxiety-craving association. The interaction between between-person anxiety and DI on craving was non-significant (*b* = 0.119, β = 0.052, *se* = 0.222, *p* = 0.592). Please see Table 1 and Fig. 1 for full results of the interaction.

## 3. Discussion

Females who smoke cigarettes are at increased susceptibility for anxiety (Mclean et al., 2012), which can exacerbate craving and reinforce smoking behavior in their daily lives. High distress intolerance (DI), a trait-level vulnerability for both anxiety (Keough et al., 2010) and smoking (Veilleux, 2019), could amplify the anxiety-craving association among this at-risk group; nevertheless, limited prospective work explores relations between these variables simultaneously. Thus, the present EMA study examined the effect of momentary anxiety on momentary cigarette craving and the moderating influence of trait DI among a sample of 50 females who endorsed daily cigarette use. Our results largely corroborate and extend prior theoretical and empirical work on the role of DI in anxiety and smoking outcomes, as discussed further below. Whereas momentary anxiety (indexed both within and between person) was consistently associated with increased cigarette craving, DI moderated the link between within-person variability in momentary anxiety and momentary craving. The absence of both a main effect of DI on momentary craving and a between-person interaction suggests a contingent role of this transdiagnostic vulnerability based on females’ emotional contexts.

Higher DI, relative to lower DI, amplified the association between within-person variability in anxiety and concurrent cigarette craving. This finding persisted while controlling for baseline anxiety, nicotine dependence, the main effect of DI, and cigarettes smoked on the prior day, underscoring the robustness of this result. Our results align with other EMA work by Volz and colleagues (2014) where the association between daily stressors and daily cigarette craving was strongest for those higher in behavioral DI. Our study extends Volz’s (2014) findings by demonstrating that increases in craving as a function of increases in anxiety are even greater for females higher in self-reported DI. This is notable given prior work finds that behavioral and self-reported DI measures are not strongly correlated (Glassman et al., 2016). Perhaps continuing to conceptualize DI as a vulnerability that is best considered in the context of acute emotional experiences is one avenue toward clarifying prior discrepant findings.

Notably, there was no main effect of DI on craving. Whereas some work has found a direct link between DI and craving (Abrantes et al., 2008; Farris et al., 2016; Trujillo et al., 2015), some has not (Lim et al., 2018; Volz et al., 2014). Thus, it is plausible that DI’s vulnerability is context-dependent, both upon *what* activates DI and *how* tolerance is being assessed. Our results suggest that DI may be a vulnerability for craving that is only “activated” in the context of experienced emotional distress (i.e., in this case, in the presence of momentary anxiety). The present findings build upon other studies showing that DI conditionally predicts nicotine withdrawal symptoms (Farris et al., 2015) and lapse during a cessation attempt (Abrantes et al., 2008) in the context of heightened negative affect. Other work shows that high DI uniquely predicts negative affect expectancies for cigarette use, but not other smoking expectancies (e.g., boredom reduction, negative social impressions, negative physical feelings; Shadur et al., 2017). Our findings also underscore that assessment of DI (i.e., domain-general versus specific) could account for discrepant results. One study found that only smoking-specific DI (i.e. intolerance of tobacco withdrawal symptoms), but not general DI (i.e. intolerance of emotional experiences), was related to cigarette craving (Mathew et al., 2019).

Aligned with these findings, Veilleux (2023) recently proposed a momentary (i.e. state-level), contextual model of distress tolerance that considers how factors like momentary distress intensity, and other experiences (e.g., social support, hunger, self-efficacy) shape behavioral responding to distress (i.e. engagement with or avoidance of distress).

Future EMA studies could build upon this theoretical work by incorporating brief assessments for intensity of other distress states (e.g. sadness, anger), psychological resources (e.g. hunger, tiredness) and contextual variables (e.g. presence of social support, physical environment) in predicting momentary distress intolerance (via the Momentary Distress Intolerance Scale (MDIS); Veilleux et al., 2018) and behavioral outcomes (e.g. use of emotion regulation strategies, substance use). Further consideration of the trait- and state-level properties of DI could bolster the emotion-dependent conditional effect of DI on smoking outcomes. Evaluation of this model in the context of cigarette use could also serve to inform tailored in-the-moment interventions for smoking cessation.

To our knowledge, this is the first EMA study to prospectively examine the effect of momentary anxiety and cigarette craving, and the moderating role of DI among females who smoke cigarettes daily. This high-definition EMA data afforded evidence of DI’s conditional effect, over and above support yielded by previous cross-sectional and experimental work. This significant moderation effect was presented above and beyond the effect of covariates, including cigarette use, trait anxiety, and cigarette dependence. Continued research using such granular data collection methods that consider emotional context have the potential to explain null and inconsistent findings noted throughout the DI and smoking literature (Veilleux, 2019). Additionally, by testing both between- and within-person hypotheses simultaneously, we were able to identify precisely when DI’s vulnerability is activated – specifically, on days in which anxiety is higher than a person’s average. In-the-moment interventions for smoking cessation might target high-anxiety days for individuals with high DI. Moreover, the present work specifically focused on females, who appear vulnerable to both anxiety pathology (Mclean et al., 2012) and heightened craving in the context of negative emotional states (Erblich et al., 2022; Saladin et al., 2012). These results highlight anxiety and DI as potential female-specific intervention targets for smoking cessation.

Several limitations are also worthy of consideration. Our assessment of DI at baseline via a single self-report measure (Simons & Gaher, 2005) taps one’s perceived ability to tolerate psychological distress, in general (i.e., “trait”). State (momentary) DI, as measured with items on the MDIS (Veilleux et al., 2018) could be used to add further granularity to the study of individuals’ emotional milieu and how DI’s fluctuations influence craving. Similarly, we indexed anxiety and craving via single items at each random report. Although seemingly face valid and used in prior EMA studies (Sandel-Fernandez et al., 2023; Veilleux & Skinner, 2019), these items preclude examination of specific facets of anxiety (e.g. source of anxiety, specific symptoms) and craving (e.g. likelihood to act on craving). Future studies might ask additional questions regarding these variables to increase nuanced understanding of these associations. Additionally, although a strength in some ways, our sample was comprised of a small, relatively homogenous group of biological females who were recruited from the community, which may limit the generalizability of findings. Examination of these associations among larger, more diverse samples is warranted, as DI and momentary experiences of anxiety and craving may vary with demographic (e.g. race, sex) and smoking characteristics (e.g. frequency, dependence level, interest in cessation). In fact, prior EMA work shows those who smoke intermittently (as opposed to daily) reported greater DI prior to smoking in the context of low positive affect (Veilleux & Skinner, 2019). Follow-up studies are needed to explore the generalizability of these findings.

It is important that research continue to leverage daily- and momentary-level prospective data to understand DI’s contextual influence on smoking-relevant processes. For instance, future work should explore whether DI exerts stronger influence in the context of nicotine withdrawal or among daily versus intermittent smokers. Such data has the potential to inform both the etiology of anxiety-smoking links as well as ideographic, in-the-moment interventions. Individuals high in DI might benefit from psychoeducation about the link between affective experiences and craving (Sala et al., 2021). In fact, more frequent monitoring of internal experiences results in less anxiety and craving, (Mccarthy et al., 2015) which could engender earlier, more adaptive responses to distress. Relatedly, the mixed success of previous DI-targeted smoking treatments (Brown et al., 2013; Farris et al., 2016; Luberto & Mcleish, 2017; Murray, 2007) could stem from suboptimal intervention timing. Providing individuals high in DI with emotion regulation or urge surfing skills to use in advance of elevated anxiety can perhaps thwart cigarette cravings and reduce likelihood of lapse. Mobile health and/or just-in-time adaptive interventions might be uniquely warranted avenues of intervention for folks with high DI in the context of the present findings. Offering real-time feedback and strategies to females high in DI skills during moments of elevated anxiety could promote better tobacco cessation outcomes.

## Figures and Tables

**Table 1:** Multilevel modeling results.

Model	Effect	Standardized Beta	Unstandardized Beta	Standard Error	*t Value*	*p*	CI(l)	CI(u)
**1**	Intercept	0.010	1.711	0.686	2.49	0.016	0.331	3.091
	Cigarette Dependence (FTCD)	0.055	0.081	0.119	0.68	0.500	−0.153	0.315
	Same-Day Cigarettes Smoked	0.055	0.030	0.017	1.74	0.083	−0.004	0.063
	Trait Anxiety (DASS-Anxiety)	0.092	0.054	0.047	1.14	0.254	−0.039	0.147
	**Anxiety**	**0.383**	**0.740**	**0.150**	**4.94**	**0<.001**	**0.446**	**1.033**
	**Momentary Anxiety**	**0.198**	**0.346**	**0.026**	**13.32**	**0<.001**	**0.295**	**0.397**
	Distress Intolerance (DTS)	0.071	0.283	0.300	0.94	0.348	−0.307	0.872
**2**	Intercept	0.022	1.840	0.721	2.55	0.014	0.389	3.290
	Cigarette Dependence (FTCD)	0.057	0.083	0.119	0.70	0.484	−0.150	0.316
	Same-Day Cigarettes Smoked	0.053	0.052	0.047	1.11	0.268	−0.040	0.145
	Trait Anxiety (DASS-Anxiety)	0.089	0.029	0.017	1.69	0.092	−0.005	0.062
	**Anxiety**	**0.368**	**0.710**	**0.158**	**4.50**	**0<.001**	**0.401**	**1.020**
	**Momentary Anxiety**	**0.188**	**0.328**	**0.026**	**12.56**	**0<.001**	**0.277**	**0.380**
	Distress Intolerance (DTS)	0.048	−0.119	0.795	−0.15	0.881	−1.678	1.441
	DTS*Anxiety	0.052	0.119	0.221	0.54	0.592	−0.316	0.553
	DTS*Momentary Anxiety	**−0.056**	**−0.117**	**0.027**	**−4.29**	**0<.001**	**−0.171**	**−0.064**

Note: Anxiety = Anxiety centered between person; Momentary Anxiety = Anxiety centered within-person; DTS = Distress Tolerance Scale; DASS-Anxiety = Depression, Anxiety, and Stress Scale - Anxiety subscale; FTCD = Fagerstrom Test of Cigarette Dependence. Bold represents significance at *p* < 0.05.

## Data Availability

Data supporting the conclusions of this manuscript will be made available by the authors upon reasonable request.
